# Sleepiness among personnel in the Norwegian Air Ambulance Service

**DOI:** 10.1007/s00420-019-01449-w

**Published:** 2019-06-10

**Authors:** Tine Almenning Flaa, Anette Harris, Bjørn Bjorvatn, Hilde Gundersen, Erik Zakariassen, Ståle Pallesen, Siri Waage

**Affiliations:** 1 Norwegian Air Ambulance Foundation, Oslo, Norway; 2 Department of Global Public Health and Primary Care, University of Bergen, Bergen, Norway; 3 Department of Psychosocial Science, University of Bergen, Bergen, Norway; 4 Norwegian Competence Center for Sleep Disorders, Bergen, Norway; 5 Department of Sport and Physical Education, Western Norway University of Applied Sciences, Bergen, Norway

**Keywords:** Sleepiness, Shift work, Air ambulance, Pilots, HEMS crew members

## Abstract

**Abstract:**

Purpose To examine the effects of shift work and extended working hours on sleepiness among pilots and Helicopter Emergency Medical Service (HEMS) crew members in the Norwegian Air Ambulance.

**Methods:**

This field study investigated sleepiness during 3 consecutive weeks: the week before work, the work week, and the week after work. The pilots and HEMS crew members (*N* = 50) kept a wake diary during all 3 weeks and completed reaction time tests during the work week.

**Results:**

The overall sleepiness scores were low during all 3 weeks. When comparing the 3 weeks, the lowest sleepiness levels were found for the work week. There was a small difference across work days, in which subjective sleepiness scores were highest the first duty day. No change in the reaction time tests was evident during the work week. The crew members reported being most sleepy at midnight, compared to all the other timepoints over the course of a duty day. Regarding workload and total work time, having larger workload was associated with lower sleepiness scores, while having higher total work time was associated with higher sleepiness score, both compared to the medium category.

**Conclusions:**

The findings indicate that the work schedules and setting for this distinct occupational group do not seem to negatively affect the sleepiness levels.

## Introduction

To maintain around the clock operations, a large portion of the workforce must be organized in shift work and often works extended hours. Shift work can generally be denoted as work outside normal daytime, and may include work during evening, night, and weekend (National Sleep Foundation 2018). Shift work, including night shift, implies activity and light exposure at a time when inactivity and rest are natural (Akerstedt and Wright 2009). Work during the natural night often influence sleep and functioning and may result in shortened sleep and/or increased sleepiness (Swanson et al. 2011). Studies generally suggest a reduction of sleep duration by approximately 3 h following night shifts (Sallinen and Kecklund 2010), and severe sleepiness has been found during night shift, as this coincides with the nadir for the core body temperature (Härmä et al. 2002; Akerstedt and Wright 2009). Night work is, therefore, associated with impaired performance and occupational accidents (Härmä and Kecklund 2010; Wagstaff and Lie 2011). In addition, working irregular shifts poses a risk for increased sleepiness and sleep debt, indicating an elevated need for recovery (Härmä et al. 2018). Extended working hours have been defined as work exceeding 48 h a week (Harrington 2001). The literature regarding the effect of such working hours on sleep and sleepiness is inconsistent. Some studies suggest that long working hours are associated with increased sleepiness and shortened sleep (Sallinen and Kecklund 2010; Swanson et al. 2011), while other studies conclude that workers adapt quite well (Bjorvatn et al. 2006; Forberg et al. 2010). A review article concluded that extended working hours increased the risk of excessive sleepiness, which further could have implications for performance on the job and injuries (Caruso 2014). Shift work and extended work hours are common in aviation. Many flight operations include long-haul flights and crossing of several time zones. However, these work conditions are quite different from those in the Helicopter Emergency Medical Services (HEMS) characterized by on-demand medical service implying low predictability of workload and work hours. The work schedule and conditions of HEMS crews vary a great deal, both between countries and agencies. Some work 12-, 24-, or 48-h shifts, while others work 7 consecutive days. Furthermore, some live on the base, while others commute home every day when off work (Radstaak et al. 2014; Sallinen et al. 2018). These differences complicate generalization of findings across various operations, schedules, and conditions. In Norway, HEMS crews work 24-h shifts over 7 consecutive days to provide around the clock coverage and operation. However, little is known about the effects this type of shift schedules and these work characteristics have on sleepiness. Sleepiness is established as a risk factor in aviation, and evidence suggests that sleepiness is associated with flying errors and accidents (Goode 2003; Previc et al. 2009). A common distinction is drawn between subjective and objective sleepiness. Objective measures of sleepiness may include measures of sleep tendency (i.e., the Multiple Sleep Latency Test), reduced activation (i.e., pupillometry), or performance deficits (i.e., reaction time). Subjective sleepiness can be subdivided into state measures, which are sensitive to abrupt changes caused, for example, by sleep deprivation, and trait measures, which primarily measures the respondents’ general tendency to experience sleepiness. There are few scientific publications concerning sleepiness in the HEMS, and the few existing findings are ambiguous. Furthermore, the quality of the existing research is often limited due to low response rate (Müller et al. 2014), and low external validity caused by variations in shift systems across countries and agencies (Guyette et al. 2013). In addition, there seems to exist a discrepancy between results resting on measures of subjective sleepiness and those reflecting performance and objective measures of sleepiness (Müller et al. 2014; van Dongen et al. 2003). Furthermore, an appraisal of the fluctuation in sleepiness is important and could have implications for safety and health, for both personnel and patients. Consequently, knowledge about the effects of working long hours and consecutive shifts on sleepiness of pilots and HEMS crew members is thus essential. This current field study was conducted among pilots and HEMS crew members working for the Norwegian Air Ambulance (NAA). The NAA operated 9 out of 12 bases in Norway, including 10 of total 13 helicopters. The specific aim of the present study was to examine sleepiness in pilots and HEMS crew members before, during, and after a work week by administering subjective as well as objective measures of sleepiness. We hypothesized that sleepiness scores (measured with the Accumulated Time with Sleepiness Scale) would be higher during the work week, compared to the week before and after work (H1). Considering the work week, we expected that sleepiness (measured with the Accumulated Time with Sleepiness Scale, the Karolinska Sleepiness Scale, reaction time, and response accuracy) would increase over the work week (H2). We also hypothesized that during a day, the crew would report more sleepiness (measured with the Karolinska Sleepiness Scale) at the end of the wake period (H3). Finally, we expected that the crew members with larger amount of work (number of missions/training sessions and total work time) on duty would report higher sleepiness scores (measured with the Accumulated Time with Sleepiness Scale and the Karolinska Sleepiness Scale) compared to those with medium work amount (H4).

## Method

### Participants

The data derived from a study among workers in the NAA. All pilots and HEMS crew members (*n* = 70) working on all nine bases operated by the NAA were invited to participate in the study which started during the fall 2014. A total of 61 pilots and HEMS crew members agreed to participate in the first data collection, yielding a response rate of 87.1%. Of all the workers who participated in the first data collection, 59 were invited to take part in a second study that took place in spring/summer of 2015. In all, 50 workers agreed to participate, yielding a response rate of 84.7%. The current study presents data from the second data collection comprising 50 pilots and HEMS crew members, as we expected more activity during the summer weeks.

### Procedure

The data collection was conducted during the spring and summer of 2015 and took part over 3 consecutive weeks: the week before work, the work week, and the week after work. The shift schedule starts with a 7-day work week followed by 14-day off duty, then 7-day on duty followed by 21-day off duty. The shift starts and ends at 10.00 on Monday morning, where the crew commutes either the day before or on the same day as the shift start. There are geographical differences between the bases, and the crew of those that are placed in rural areas often need to commute over longer distances. The crew operates missions both at day and night throughout the year. During the work week, the crew lives together on the bases with all necessary facilities, including separate bedrooms, exercise room, kitchen, and a living area with TV. The fatigue risk management system includes flight and work limits, divided into flight time and active work time approved by the Civil Aviation Authority of Norway. Flight time refers to time spent in the helicopter and active work time starts when an alarm goes off and ends 1 h after completed flight. Maximum flight time is 7 h in a consecutive 24 h period, 12 h in a consecutive 48 h period, and 30 h in a 7-day period. The maximum active work time is 14 h during a consecutive 24 h period and 30 h during a consecutive 72-h period. If the crew reaches a limit, they need to go off flight duty for 8 h before returning. In addition to providing medical service by helicopter, the HEMS crew members sometimes drive a rapid response car to close-by locations. The workers completed a questionnaire on their first duty day. Furthermore, they kept wake diaries for the 3 weeks and performed a reaction time test several times during the work week. The study included data from the mission log reported by the bases.

### Instruments

#### Questionnaire

The questionnaire included demographic and background variables such as sex, age, marital status, and children living at home. It also covered questions about sleep need, sleep problems related to work schedule (ranging from none to very much), degree of sufficient sleep at work (ranging from never to very often), frequency of work weeks with less than 5 h of sleep (ranging from the occasional work week to every work week), caffeine, nicotine, commute, and second jobs.

#### Epworth Sleepiness Scale (ESS)

The ESS (Johns 1991) is an 8-item scale that measures the subject’s general tendency to sleep or doze off in eight different situations. The scale is thus a subjective trait measure of sleepiness with a 4-point scale, yielding a total score between 0 and 24. Scores higher than 10 indicate excessive daytime sleepiness. The ESS demonstrated good internal consistency with an alpha reliability at 0.84. The ESS was administered once, on the first duty day.

#### Mission log

An overview of all the missions, training sessions, and the amount of time spent in active work was provided by the NAA. The workload variable included both missions and training sessions. Total work time (TWT) was calculated from the time the alarm went off to the time they landed after a mission. If a training session was conducted, the TWT was calculated from when the training session started to the time they landed back at the base. Based on tertiles, categorical variables were made for workload and TWT. Night work was defined as mission taking place between 24:00 and 07:00, including those missions that started before midnight and ended after midnight.

#### Sleepiness measured with wake diary

The Accumulated Time with Sleepiness (ATS) scale (Gillberg et al. 1994) is designed as a method for integrating measures of subjective sleepiness over longer time periods. Occurrence and duration (proportion of the wake period when the symptom was present, ranging from 0 to 100%) of specific symptoms of sleepiness during the wake period are rated. Six items were used in the present study, including “heavy eyelids”, “feeling gravel-eyed”, “difficulty in focusing your eyes”, “irresistible sleepiness”, “reduced performance”, and “periods where you were fighting sleep”. The scale is regarded as a state measure of sleepiness. Mean scores of week and days were calculated for each item, and a good internal consistency was demonstrated with an alpha reliability at 0.92. The ATS was sent by postal mail to the participant’s home and was administered every day, before bedtime, for all 3 weeks.

The Karolinska Sleepiness Scale (KSS; Akerstedt and Gillberg 1990) consists of a 9-point graded, scale measuring subjective sleepiness rated from 1 = very alert, 3 = alert, 5 = neither alert nor sleepy, 7 = sleepy but no problems staying awake to 9 = very sleepy, fighting sleep, effort to stay awake. A score of seven or more indicates excessive sleepiness. The scores 2, 4, 6, and 8 are not verbally anchored. The KSS assesses state sleepiness and the participants completed the KSS every other hour while awake during the work week. Mean scores were calculated for each duty day and every other hour awake during the duty days.

#### Sleepiness measured with reaction time test

A task based on the Posner-cue-target paradigm was included as an objective measure of reaction time, inhibition and accuracy (Gundersen et al. 2007; Posner and Driver 1992) and was programmed using the standard version of E-Prime 2.0. (Psychology Software Tool). The task was administered on a laptop and the participants were instructed to complete the test while sitting down in a comfortable position, in quiet surroundings. During testing, the participants were told to fixate on a crosshair between two rectangular frames on the screen. When a target stimulus appeared in either of the frames, the participants were instructed to hit the ‘D’ (when stimulus appeared in left frame) or ‘L’ (when stimulus appeared in right frame) on the keyboard as fast as they could. The frames would sometimes be broadened (i.e., a cue) before the target stimulus appeared, which the participants were told to ignore. There were three categories incorporated in the test: “no cue”, “valid cue”, and “invalid cue”. “No cue” implied that the target stimulus appeared in one of the frames without any cue. In the “valid cue” category, the target stimulus appeared in a broadened frame, while in the “invalid cue” category, the target stimulus appeared in the opposite to the broadened frame. The test lasted for 4 min and 40 s and 168 target stimuli were presented during each test session. Each of the target stimuli was presented for 500 ms and the rest intervals between each stimulus were randomized and lasted for 600–1400 ms. The cue appeared 200 ms or 400 ms before the target stimulus was presented. The distribution of the target stimuli was 16.7% for “no cue”, 16.7% for “invalid cue”, and 66.6% for “valid cue”. Reaction time (RT) and response accuracy (RA) for each category were calculated. The participants completed the test five times during the work week: in the evening the first day at work, in the morning at midweek, in the evening at midweek, in the morning at the end of the work week, and in the evening at the end of the work week. They were instructed to take the evening tests right before bedtime and the morning tests within an hour after wake time.

### Data analyses

All statistical analyses were conducted using SPSS version 25. A linear mixed model approach was applied to produce unbiased estimates of variance and covariance parameters (West et al. 2014). In the analysis of ATS over the 3 weeks, week was included as a fixed factor, where the second week (work week) was set as reference. The analysis of ATS during the work week included day, workload, and TWT as fixed factors. The analysis of KSS during the work week included day, time of day, workload, and TWT as fixed factors. At day 1, 24:00 in the time of day variable, medium workload, and medium TWT were set as reference categories in the two latter analyses. The effect of bases was adjusted for in the analysis. In the analysis of reaction time, fixed effect for test points was modelled. Subjects were included as a random factor in all analyses. Alpha values less than 0.05 were considered as statistically significant.

### Missing data

On the six items from the ATS scale, missing data comprised 8.8% of the total in heavy eyelids, 8.8% of feeling gravel-eyed, 9.0% of difficulty in focusing your eyes, 8.9% of irresistible sleepiness, 8.7% of reduced performance, and 8.8% of periods, where you were fighting sleep. The proportion of missing data on the KSS was 6.5%, and for reaction time test, the proportion of missing data was 43.2% (108 out of 250 individual tests). Data on the reaction time test were missing at random, with no evident pattern in terms of distribution around timepoints.

## Results

### Descriptive statistics

Twenty-five pilots and 25 HEMS crew members participated in the study, representing nine different bases across Norway. In all, the sample consisted of 49 (98.0%) males and one female (2.0%). The mean age was 43.8 years (SD = 7.2), range 29–59 years. In all, 90.0% (*n* = 45) were married or cohabiting and 78.0% (*n* = 39) had children living at home. A total of 86.0% (*n* = 43) reported getting less than 5 h of sleep on duty occasionally or sometimes, 92.0% (*n* = 46) reported little or no problems related to sleep at work, and 80.0% (*n* = 40) reported getting enough sleep on duty. Data on intake of caffeine, use of nicotine, and characteristics about their commute are presented in Table 1. The mean ESS score was 7.1 (SD = 3.9). In all, 20 workers had a second job with a mean employment percentage of 22.4% (SD = 14.86), range 2–50%. During the work week, mean workload (number of missions and training sessions combined) was 17.3 (SD = 6.1), ranging from 5 to 30. Of these, 1.4 (SD = 1.0) was night work ranging from 0 to 4 during the work week. The mean TWT was 25.2 h (SD = 8.8) during the work week. TWT ranged from 7.1 to 48.0 h.

**Table 1:** Descriptive statistics regarding means of caffeine and nicotine consumptions, commute, commute length, and day of commute, reported in terms of number of participants (*n*) and percentages (%) among pilots and HEMS crew members in Norway (*N* = 50)

	*n* (%)
Caffeine on duty^a^	
No caffeine	2 (4.1)
1–2 cups	2 (4.1)
3–6 cups	35 (71.4)
≥ 7 cups	10 (20.4)
Smoke	
Yes	0 (0.0)
No	48 (100.0)
Snuff	
Yes	7 (14.6)
No	41 (85.4)
Means of commute	
Walk/bicycle	3
Car	34
Bus/train	16
Plane	18
Other	4
Commute length	
< 1 h	14 (29.8)
1–3 h	16 (34.0)
3–6 h	15 (31.9)
6–12 h	2 (4.3)
Commute day	
The day before first duty	1 (2.1)
The same day as first duty	46 (97.9)

^a^Cups of coffee, tea, cola, or energy drink, containing caffeine

### Sleepiness measured with wake diary

#### Sleepiness measured with Accumulated Time with Sleepiness (ATS) across 3 weeks

There were significant main effects of week on all six ATS components: heavy eyelids *F* (2, 907) = 10.1, *p* < 0.001 [estimated marginal means (SEM) week 1: 9.06 (1.76), week 2: 5.61 (1.75), week 3: 8.31 (1.78)], feeling gravel-eyed *F* (2, 911) = 4.81, *p* < 0.01 [estimated marginal means (SEM) week 1: 3.49 (0.983), week 2: 2.07 (0.981), week 3: 3.69 (1.0)], difficulty in focusing your eyes *F* (2, 910) = 5.01, *p* < 0.01 [estimated marginal means (SEM) week 1: 2.58 (0.779), week 2: 1.32 (0.778), week 3: 2.70 (0.795)], irresistible sleepiness *F* (2, 906) = 5.37, *p* < 0.01 [estimated marginal means (SEM) week 1: 5.54 (1.53), week 2: 3.44 (1.53), week 3: 5.50 (1.55)], reduced performance *F* (2, 916) = 13.9, *p* < 0.001 [estimated marginal means (SEM) week 1: 4.67 (0.759), week 2: 1.78 (0.756), week 3: 3.76 (0.781)], and periods where you were fighting sleep *F* (2, 907) = 6.28, *p* < 0.01 [estimated marginal means (SEM) week 1: 5.67 (1.65), week 2: 3.76 (1.65), week 3: 5.96 (1.67)], see Table 2 for estimates. When comparing HEMS crew members (M = 1.67, SD = 6.09) and pilots (M = 2.82, SD = 9.47), the pilots had slightly higher scores on the ATS item “difficulty in focusing your eyes” (*p* < 0.05).

**Table 2:** Effects of weeks on subjective sleepiness, measured by the Accumulated Time with Sleepiness (ATS), across 3 weeks among pilots and HEMS crew members in Norway (*N* = 50)

	Week 1	Week 2^a^	Week 3
	Estimate (SEM)		Estimate (SEM)
ATS			
Heavy eyelids	3.45 (0.804)***		2.70 (0.847)**
Feeling gravel-eyed	1.42 (0.558)*		1.62 (0.589)**
Difficulty in focusing your eyes	1.27 (0.476)**		1.38 (0.501)**
Irresistible sleepiness	2.09 (0.722)**		2.06 (0.761)**
Reduced performance	2.89 (0.559)***		1.98 (0.589)**
Periods, where you were fighting sleep	1.91 (0.661)**		2.20 (0.696)**

Results are from linear mixed models including multilevel estimates and standard error of the means (SEM)

*p < 0.05, **p < 0.01, ***p < 0.001

^a^The second week (work week) represents the reference group

#### Sleepiness measured with Accumulated Time with Sleepiness (ATS) during work week

There were no significant differences in the sleepiness scores for the six ATS components during the work week. Neither workload nor TWT affected these scores. The main effects of day on each ATS component were: heavy eyelids *F* (6, 276) = 0.419, *p* = 0.866, feeling gravel-eyed *F* (6, 277) = 0.707, *p* = 0.645, difficulty in focusing your eyes *F* (6, 278) = 0.823, *p* = 0.553, irresistible sleepiness *F* (6, 274) = 1.55, *p* = 0.161, reduced performance *F* (6, 281) = 1.14, *p* = 0.340, and periods where you were fighting sleep *F* (6, 276) = 1.59, *p* = 0.150. The main effects of workload on each ATS component were: heavy eyelids *F* (2, 284) = 1.75, *p* = 0.175, feeling gravel-eyed *F* (2, 296) = 1.69, *p* = 0.186, difficulty in focusing your eyes *F* (2, 303) = 2.91, *p* = 0.056, irresistible sleepiness *F* (2, 280) = 1.55, *p* = 0.215, reduced performance *F*(2, 312) = 1.81, *p* = 0.166, and periods where you were fighting sleep *F* (2, 282) = 2.31, *p* = 0.101. The main effects of TWT on each ATS component were: heavy eyelids *F* (2, 284) = 0.201, *p* = 0.818, feeling gravel-eyed *F* (2, 296) = 0.010, *p* = 0.990, difficulty in focusing your eyes *F* (2, 302) = 0.186, *p* = 0.830, irresistible sleepiness *F* (2, 280) = 0.024, *p* = 0.977, reduced performance *F* (2, 310) = 0.041, *p* = 0.960, and periods where you were fighting sleep *F* (2, 282) = 0.089, *p* = 0.915, see Table 3 for estimated marginal means.

**Table 3:** Estimated marginal means (M) and standard error of the mean (SEM) on the Accumulated Time with Sleepiness (ATS) across 7 work days, workload and total work time (TWT) among pilots and HEMS crew members in Norway (*N* = 50)

	Day M (SEM)							Workload M (SEM)			TWT M (SEM)		
	1	2	3	4	5	6	7	Lower	Medium	Higher	Lower	Medium	Higher
ATS													
1	5.21 (2.06)	4.71 (2.02)	6.15 (2.01)	5.97 (2.02)	5.48 (1.99)	7.11 (2.02)	4.59 (2.00)	7.29 (1.69)	5.55 (1.94)	3.97 (1.97)	5.19 (1.98)	5.41 (1.74)	6.21 (1.77)
2	2.42 (1.15)	0.687 (1.11)	2.35 (1.10)	1.60 (1.11)	1.14 (1.09)	2.98 (1.11)	1.54 (1.10)	2.96 (0.801)	1.63 (1.03)	0.774 (1.06)	1.78 (1.07)	1.86 (0.857)	1.73 (0.874)
3	1.21 (0.923)	− 0.104 (0.881)	1.36 (0.870)	0.638 (0.876)	0.645 (0.866)	2.20 (0.873)	0.653 (0.864)	2.30 (0.583)	0.554 (0.810)	-0.024 (0.825)	0.681 (0.830)	0.871 (0.641)	1.28 (0.651)
4	1.25 (1.93)	2.84 (1.90)	4.92 (1.89)	3.64 (1.90)	3.90 (1.88)	5.53 (1.89)	2.71 (1.88)	5.00 (1.65)	2.97 (1.83)	2.66 (1.87)	3.47 (1.87)	3.68 (1.69)	3.48 (1.71)
5	1.76 (0.863)	0.717 (0.833)	2.67 (0.823)	0.828 (0.829)	1.27 (0.810)	2.61 (0.817)	0.960 (0.817)	2.59 (0.523)	1.16 (0.749)	0.886 (0.770)	1.39 (0.774)	1.59 (0.582)	1.66 (0.593)
6	1.14 (1.93)	3.37 (1.90)	4.59 (1.89)	4.19 (1.89)	4.21 (1.88)	5.53 (1.88)	2.73 (1.88)	5.40 (1.66)	3.20 (1.84)	2.44 (1.86)	3.48 (1.87)	3.94 (1.70)	3.62 (1.72)

*ATS 1* heavy eyelids, *ATS 2* feeling gravel-eyed, *ATS 3* difficulty in focusing your eyes, *ATS 4* irresistible sleepiness, *ATS 5* reduced performance, *ATS 6* periods were you were fighting sleep

#### Sleepiness measured with the Karolinska Sleepiness Scale (KSS) during work week

There were significant main effects on the KSS for day *F* (6, 2539) = 4.66, *p* < . 001, time of day *F* (8, 2535) = 49.61, *p* < 0.001, workload *F* (2, 2561) = 4.93, *p* < 0.01, and TWT *F* (2, 2559) = 4.48, *p* < 0.05. Day 1 had higher scores compared to the remaining 6 days. For time of day, the KSS scores were significantly higher at midnight (24:00) compared to all the other hours. Those with higher workload reported lower KSS scores compared to those with medium work load, while those with higher TWT reported higher KSS scores compared to those with medium TWT, see Table 4 for estimates and estimated marginal means. There was no significant difference between the scores for the HEMS crew members and pilots (*p* = 0.06).

**Table 4:** Effects of days (1–7), time of days (08:00–24:00), workload, and total work time (TWT) during the work week for subjective sleepiness, measured by the Karolinska Sleepiness Scale (KSS) among pilots and HEMS crew members in Norway (*N* = 50)

	Estimate (SEM)	M (SEM)
Days		
1^a^		3.23 (0.165)
2	– 0.464 (0.096)***	2.76 (0.165)
3	– 0.283 (0.098)**	2.94 (0.164)
4	– 0.405 (0.097)***	2.82 (0.164)
5	– 0.342 (0.098)***	2.88 (0.164)
6	– 0.285 (0.103)**	2.94 (0.164)
7	– 0.316 (0.102)***	2.91 (0.165)
Time		
08	– 0.751 (0.136)***	3.38 (0.183)
10	– 1.50 (0.116)***	2.63 (0.169)
12	– 1.77 (0.112)***	2.34 (0.166)
14	– 1.77 (0.111)***	2.35 (0.166)
16	– 1.53 (0.111)***	2.57 (0.166)
18	– 1.27 (0.112)***	2.84 (0.166)
20	– 1.39 (0.111)***	2.74 (0.166)
22	– 0.880 (0.111)***	3.25 (0.166)
24^a^		4.13 (0.170)
Workload		
Low	0.096 (0.086)	3.06 (0.154)
Medium^a^		2.96 (0.162)
High	– 0.194 (0.089)*	2.77 (0.163)
TWT		
Low	– 0.081 (0.079)	2.81 (0.163)
Medium^a^		2.89 (0.156)
High	0.185 (0.074)*	3.08 (0.156)

Results are from linear mixed models including estimates, standard error of the mean (SEM) and estimated marginal means (M)

Adjusted for the effect of base

The KSS scores are based on means for each day and each hour

*p < 0.05, **p < 0.01, ***p < 0.001

^a^Day 1, time 24:00, medium workload, and medium TWT represents the reference groups

### Sleepiness measured with reaction time test

#### Reaction time test (response time and response accuracy) during work week

There was no significant main effect of time for RT_no cue_*F* (4, 98) = 1.58, *p* = 0.186, RT_valid cue_*F* (4, 98) = 1.30, *p* = 0.276, and RT_invalid cue_*F* (4, 98) = 0.972, *p* = 0.427, across five test points over the work week. There was no significant main effect of time for RA_no cue_*F* (4, 98) = 0.407, *p* = 0.803, RA_valid cue_*F* (4, 98) = 1.84, *p* = 0.127, and RA_invalid cue_*F* (4, 98) = 0.600, *p* = 0.664 across five test points over the work week.

## Discussion

The sleepiness scores measured by the ATS were lowest in the work week, compared to the weeks at home before and after work. During the work week, the highest sleepiness scores measured by the KSS were reported on the first day of work. However, there was no change during the week in terms of reaction time and response accuracy. Over the course of the day, the highest sleepiness scores measured by the KSS were reported at midnight. Having higher workload was associated with lower sleepiness measured by the KSS compared to medium workload, whereas having longer TWT was associated with higher sleepiness scores measured by the KSS, compared to a medium TWT. The crew members felt less sleepy during their work week, compared to their weeks off (both before and after). Consequently, there was no support for the first hypothesis stating that sleepiness scores would be higher during the work week, compared to the week before and the week after work. We expected opposite findings in line with other studies (Mullins et al. 2014; Akerstedt and Wright 2009). However, the crew in the present study was living at the base during the work week which relieves them of social and domestic obligations. With available base facilities such as separate bedrooms, an exercise room and a living room, the crew likely gets sufficient rest and leisure between the missions and training sessions. This may explain the lower sleepiness scores. Excessive sleepiness during free days has been found in other studies among shift workers (Härmä et al. 2018), suggesting that accumulated sleep deprivation during the work period may become manifest on days off work. However, these studies are only partially comparable to the present due to differences in sample population and work schedule predictability. In addition, the current study’s sample had a minimum of 14-day off between the work weeks. This means that the crew already had at least 1-week off work before the first assessment week in this study. Still, higher sleepiness score compared to the work week was found, although overall sleepiness levels at all 3 weeks were low. This indicates that the slightly higher sleepiness scores in the first week, stems from sources other than work. When at home, the workers have domestic obligations, including children to take care of which could be a possible factor explaining these scores. In compliance with this, Gregory et al. (2010) found that 26% of air medical pilots reported child care as a factor that affected the ability to sleep. Furthermore, some of the crew holds second jobs during their weeks off, which could explain why the sleepiness scores are slightly higher the weeks off duty. Nevertheless, it is important to emphasize that the overall sleepiness scores across all 3 weeks were low considering that the ATS scale range from 0 to 100. Hence, although higher, the sleepiness the week before and after work was not deemed clinically elevated. A comparison between the pilots and the HEMS crew members revealed a somewhat higher score for the pilots on the “difficulty in focusing your eyes” item. As the HEMS crew members are more likely to have a higher workload due to accompanying on the rapid response car, this result seems thus reasonably. However, both the scores were low indicating that neither pilots nor HEMS crew members experienced much sleepiness across the 3 weeks. The crew reported the highest sleepiness scores on the first day at work, compared to the following 6 duty days. Furthermore, the reaction time tests did not change over the course of the work week. Given these results, the second hypothesis must be rejected, postulating that subjective and objective measures of sleepiness would increase during the work week. As the hypothesis suggested, one would expect that the crew members became sleepier over the course of the work week, due to accumulated sleep deprivation caused by shift work and the work load itself (Akerstedt and Kecklund 2005). However, there are other studies, indicating that the workers adapt to shift work during the work period. Bjorvatn et al. (2006) found a decrease in sleepiness scores, both subjective and objective, over a week of night shift offshore. However, these results could be explained by a shift in the circadian rhythm due to the week of night work. Based on the mission log in the present study, it is evident that most of the missions took place during daytime and a circadian alteration is not likely, although this should be investigated in future studies. Despite the fact that the study of Bjorvatn and colleagues comprised oil rig workers who worked a week of night shift followed by a week of day shift, that occupational group still has some similarities to our sample, such as work facilities. The offshore workers live on the oil rig during their work period, and they work shift and have extended work hours. These results could suggest that the work facilities affect sleepiness levels in a positive way during the work period, despite having work schedules that often have been reported to impact sleepiness negatively. One possible explanation for the higher sleepiness scores on the first duty day could also be related to commuting. The majority of the crew reported using a car as a commuter and using 1–6 h to commute. Furthermore, almost all commuted the same day as the shift started. As the shift started at 10.00 in the morning and having up to 6 h of commute by car on the same day, it would imply that the workers needed to wake up very early at the first work day. For this reason, commute length could explain why the workers display higher sleepiness scores on the first duty day. This should receive some attention, as subjective sleepiness is associated with an increase in automobile accidents (Bioulac et al. 2017). Another possible explanation to our findings is that phase delay due to late bedtime and rise time might have occurred during the preceding weekend (Yang et al. 2001). Nevertheless, the subjective sleepiness scores were all distributed on the lower part of the scale; thus, statistically significant results must be interpreted with prudence. The practical meaning of this result should also be considered with caution, as the difference from the remaining duty days was small. The crew members showed no evidence of increased sleepiness over the course of the work week, as measured with reaction time tests. Interestingly, there was no increase on the first duty day despite having higher subjective sleepiness scores. In accordance with other studies, this could suggest that alertness is maintained, by keeping low response time and high response accuracy, despite reporting subjective sleepiness (Cullip et al. 2014; Thomas et al. 2006). The previous studies report a decrease in performance due to sleepiness (Myers et al. 2017), while others report no difference in sleepiness and/or performance despite working long shifts (Amann et al. 2014; Guyette et al. 2013). The results from the current study provide support for the latter findings. However, it is worth mentioning that the test could activate the participants by being a distinct new component in their work environment and explain the lack of change in reaction time and response accuracy. Over the course of a duty day, the crew members did report changes in levels of sleepiness. The highest sleepiness scores were not surprisingly reported at midnight, significantly different from the other timepoints of the day. Therefore, the third hypothesis, stating that crew would experience increased sleepiness at the end of the wake period, was supported. Still, the average sleepiness scores were low and distributed between the ‘very alert’ and ‘neither sleepy nor alert’ step of the scale. The distribution of the sleepiness scores resembles an oscillation in sleepiness that follows the circadian rhythm, rather than sleepiness due to work schedule (Borbély et al. 2016). The previous studies on shift work have indicated that night work and morning work are associated with sleepiness during the day, and that rotational work, rather than fixed work, is associated with higher sleepiness (Thun et al. 2016). Furthermore, a study on air plane operations found that sleepiness levels increased after flight duty (Yen et al. 2009). The work characteristics of the air ambulance service involve both night work and early morning work, often in a rotational manner, which make the present study relevant. An interpretation of the present result is that the work schedule did not affect the sleepiness score over the course of a duty day. Two variables were created based on the mission log: workload (total number of missions and training sessions during the work week), and TWT (total time in hours and minutes spent actively working during the 7-day shift). Both were made categorical and based on tertiles. Having higher workload was associated with lower sleepiness scores compared to having medium workload. In contrast, having higher TWT was associated with higher sleepiness scores compared to medium TWT. This gives partial support for the fourth hypothesis, postulating that the crew members with larger amount of work (both workload and TWT) would have higher sleepiness scores compared to those with medium work amount. These results may indicate that the activation related to more missions reduced sleepiness levels, while the activation related to longer missions did not. This is in accordance with other studies, where higher sleepiness occurred on the longest missions during a day (Amann et al. 2014; Powell et al. 2008). Nevertheless, again, the sleepiness was overall low, indicating that the crew and pilots were sufficiently alert.

Overall, the results suggest that the crew members experienced low levels of state sleepiness despite having 7-day shift on the base and unpredictable working hours. Furthermore, the trait measure of sleepiness revealed low levels (ESS = 7.1), compared to the Norwegian male population, where the mean ESS score is 7.4 (Pallesen et al. 2007). The results obtained on the state measures of sleepiness were all within the non-pathological/non-problematic level. This is in agreement with studies from other fields that share similar work schedules and arrangements, including offshore workers, tunnel workers, and construction workers (Bjorvatn et al. 2006; Forberg et al. 2010; Persson et al. 2006).

### Strengths and limitations

Some strengths and limitations of the present study should be noted. The use of both wake diaries and reaction time tests to assess sleepiness constitutes an asset. Objective measurement enabled a higher control of motivational effects that is associated with self-report. As studies suggest, there exists some discrepancies in awareness of sleepiness levels and results of objective tests, this was also taken into consideration as both subjective and objective measures of sleepiness was included (Myers et al. 2017; van Dongen et al. 2003). Another strength was related to the two types of subjective sleepiness scales that were administered. The ATS assesses sleepiness retrospectively, while the KSS is administered in situ. In situ questionnaire ensures a more accurate rating of sleepiness by avoiding biases, such as memory bias. Furthermore, it was also an asset having both a global measure of a day (ATS) and time-specific measures throughout the day (KSS). These factors could explain why we with the ATS did not find any difference in sleepiness throughout the work week, while this was detected with the KSS. Finally, the length of the study and amount of data collected per participant allowed for a comprehensive assessment of fluctuation in the dependent variables. Regarding the analysis, the use of linear mixed models approach enabled use of available data for units where timepoint data were missing, which represents a statistical advantage. Field studies provide insight relevant to groups that are not easily accessible. These studies have limitations regarding control and sample size, but represent a strength in terms of knowledge of groups that are of specific operational interest. Finally, another strength was related to the response rate, which was high. The findings in the current study could be vulnerable to the « healthy worker effect » , as the sample reflects a group that assumingly cope well and prefer a shift work setting as workers not coping well are assumed to not initiate or to be selected out of shift work by time. Given the health and educational requirements needed to fulfill the work demands in the present occupations, these workers are even more strongly selected. The results could consequently reflect the characteristics of this group rather than the effect of the work schedule. Furthermore, it would be preferable with a larger sample size, especially for the objective data. The generalizability represents a limitation that applies for field studies in general; thus, both the low sample size and the highly selected occupational group make the results difficult to generalize to other occupational groups. However, the results could be of interest to similar groups from the same occupation in general or for other groups that share the same type of work schedule. Due to some missing data on the reaction time test, the amount of registrations on each occasion was quite limited which could affect the results. However, it is challenging to conduct field studies on such selected groups due to the special work setting and the unpredictable work sessions. Objective measures such as reaction time tests could, therefore, have been given lower priority. Future studies should focus on adaptable means of securing higher participation regarding objective testing in occupational settings. However, the total number of reaction time tests in the present study was still adequate, with 142 out of 250 tests completed. In addition, studies on sleep deprivation show that the effect of the deprivation increases, as the length of the test increases (Lo et al. 2016). The test length of 4 min and 40 s may, therefore, have been too short to reveal any real impact of sleepiness. The study lacks injury and accident data on HEMS operations, which could serve as an objective measure of sleepiness in certain incidents. In addition, the findings should also be interpreted in light of the special context (e.g., work schedule and job characteristics) and study limitations (e.g., selection bias). Future studies on this topic should assess and adjust for chronotype in the analyses.

## Conclusion

The present study revealed that the overall sleepiness scores were low during all 3 test weeks. When comparing the 3 weeks, the lowest sleepiness levels were found for the work week. There was a small difference across work days, in which subjective sleepiness scores were highest the first duty day. No change in the reaction time tests was evident during the work week. The crew members reported being most sleepy at midnight, compared to all the other timepoints over the course of a duty day. Regarding workload and TWT, having larger workload was associated with lower sleepiness scores, while having higher TWT was associated with higher sleepiness score, both compared to the medium category. To the author’s knowledge, this study is unique in being one of the first field studies on this occupational group that included both subjective and objective measures of sleepiness over an extended period. Overall, our findings indicate that the work setting and schedules for this particular occupational group do not seem to negatively affect the sleepiness levels.
